# Endothelial HSPA12B Exerts Protection Against Sepsis-Induced Severe Cardiomyopathy via Suppression of Adhesion Molecule Expression by miR-126

**DOI:** 10.3389/fimmu.2020.00566

**Published:** 2020-04-29

**Authors:** Xia Zhang, Xiaohui Wang, Min Fan, Fei Tu, Kun Yang, Tuanzhu Ha, Li Liu, John Kalbfleisch, David Williams, Chuanfu Li

**Affiliations:** ^1^Department of Surgery, James H. Quillen College of Medicine, East Tennessee State University, Johnson City, TN, United States; ^2^The Center of Excellence in Inflammation, Infectious Disease and Immunity, James H. Quillen College of Medicine, East Tennessee State University, Johnson City, TN, United States; ^3^Department of Geriatrics, The First Affiliated Hospital of Nanjing Medical University, Nanjing, China; ^4^Biometry and Medical Computing, James H. Quillen College of Medicine, East Tennessee State University, Johnson City, TN, United States

**Keywords:** endothelial HSPA12B, polymicrobial sepsis, cardiomyopathy, exosomes, microRNAs, endothelial adhesion molecules

## Abstract

Heat shock protein A12B (HSPA12B) is predominately expressed in endothelial cells (ECs) and has been reported to protect against cardiac dysfunction from endotoxemia or myocardial infarction. This study investigated the mechanisms by which endothelial HSPA12B protects polymicrobial sepsis–induced cardiomyopathy. Wild-type (WT) and endothelial HSPA12B knockout (HSPA12B^–/–^) mice were subjected to polymicrobial sepsis induced by cecal ligation and puncture (CLP). Cecal ligation and puncture sepsis accelerated mortality and caused severe cardiac dysfunction in HSPA12B^–/–^ mice compared with WT septic mice. The levels of adhesion molecules and the infiltrated immune cells in the myocardium of HSPA12B^–/–^ septic mice were markedly greater than in WT septic mice. The levels of microRNA-126 (miR-126), which targets adhesion molecules, in serum exosomes from HSPA12B^–/–^ septic mice were significantly lower than in WT septic mice. Transfection of ECs with adenovirus expressing HSPA12B significantly increased miR-126 levels. Increased miR-126 levels in ECs prevented LPS-stimulated expression of adhesion molecules. *In vivo* delivery of miR-126 carried by exosomes into the myocardium of HSPA12B^–/–^ mice significantly attenuated CLP sepsis increased levels of adhesion molecules, and improved CLP sepsis–induced cardiac dysfunction. The data suggest that HSPA12B protects against sepsis-induced severe cardiomyopathy via regulating miR-126 expression which targets adhesion molecules, thus decreasing the accumulation of immune cells in the myocardium.

## Introduction

Sepsis is defined as a life-threatening organ dysfunction caused by a dysregulated host innate and inflammatory responses to the infection (1). In the United States, the mortality rates of sepsis is 28.3%, which is higher than other disease in intensive care units (2). Cardiovascular dysfunction is a major complication associated with sepsis-induced morbidity and mortality [7;9]. Cardiomyopathy is present in >40% of sepsis patients (3, 4) and is associated with mortality rates of up to 70% (3, 4). Despite the severity of this condition, the mechanisms that mediate septic cardiomyopathy remain unclear.

Endothelial cell (EC) dysfunction contributes to multiple organ damage and high morbidity and mortality in sepsis/septic shock (5). Increasing evidence shows that ECs actively participate in both innate and adaptive immune responses (6, 7) via pattern recognition receptors, including Toll-like receptors (8). Pathogen-associated molecular patterns, such as LPS or endogenous ligands, generated during sepsis/septic shock stimulate EC activation. Activated ECs have upregulated expression of chemokines and adhesion molecules, which attract and promote immune cell infiltration and inflammatory response, resulting in organ injury (9). Therefore, preservation of endothelial function is an important approach for attenuating sepsis-induced outcomes.

HSPA12B is the newest member of the HSP70 family of proteins (10). It is predominantly expressed in ECs (11, 12) and is essential for angiogenesis (12). Stegall et al. have demonstrated that endothelial HSPA12B is involved in angiogenesis through the turnover of a known angiogenesis regulator, a kinase anchoring protein 12 (AKAP12), resulting in upregulation of VEGF expression (12). Hu et al. (11) reported the endothelial HSPA12B is involved in regulating EC function. Knockdown of HSPA12B by small interfering RNAs in human umbilical vein ECs (HUVECs) interfered with wound healing, EC migration, and tube formation. In contrast, overexpression of HSPA12B enhanced migration of ECs and accelerated wound healing (11). We have reported that transgenic mice overexpressing HSPA12B (HSPA12B Tg) exhibit protection against myocardial ischemic injury and attenuate LPS-induced cardiac dysfunction (13). HSPA12B has been reported to preserve EC function (14, 15). However, the mechanisms by which HSPA12B preserves EC function during sepsis are still unknown.

MicroRNAs (miRs) have been identified as novel regulators of gene expression at the posttranscriptional level by binding to target messenger RNAs (16, 17). Recent evidence suggests that miRs play a critical role in sepsis/septic shock–induced innate immune and inflammatory responses (16, 17). MicroRNA-126 (miR-126) is predominantly expressed in ECs (18) and has been reported to regulate the progression of angiogenesis (19) and the expression of vascular cell adhesion molecule 1 (VCAM-1) (20). MicroRNA-126 is also involved in regulation of survival and function of plasmacytoid dendritic cells via the VEGFR2 pathway (21), indicating that miR-126 may regulate innate immune responses. However, the role of miR-126 in sepsis-induced cardiomyopathy has not been investigated.

The present study has shown that endothelial-specific HSPA12B exerts a protective effect on sepsis-induced cardiomyopathy. We demonstrated that EC HSPA12B could regulate miR-126 expression, which targets adhesion molecules, resulting in decreases in the accumulation of immune cells in the myocardium, thus attenuating sepsis-induced cardiac dysfunction.

## Materials and Methods

### Animals

Male C57BL/6 mice were obtained from Jackson Laboratory. Endothelial cell–specific HSPA12B knockout (HSPA12B^–/–^) mice were generated as described below. Wild-type (WT) and HSPA12B^–/–^ mice were maintained in the Division of Laboratory Animal Resources, East Tennessee State University (ETSU). The experiments outlined in this article conform to the Guide for the Care and Use of Laboratory Animals published by the National Institutes of Health (NIH Publication, eighth edition, 2011). The animal care and experimental protocols were approved by the ETSU Committee on Animal Care.

### Generation of EC-Specific HSPA12B Knockout Mice

The knockout targeting strategy is outlined in Supplementary Figure S1. LoxP sites flanking exon 2 were introduced using a recombineering-based approach for making linearized targeting construct. The targeting construct contained PGK-driven Neo cassette and MC1 promoter–driven HSV-TK cassette, allowing for positive and negative selection. The right and the left arm loxP knockin were confirmed by genomic Southern blot with (*Eco*RI and probe A) and *Sal*l digestion of polymerase chain reaction (PCR) product by external and internal primer (5′-TCTGTGTCTGCCTGTGTTCTGT and 5′-TAGTCTGCATTCGGAGGCAAGT). The successful homologous recombination clones were subsequently transfected with pCre-Pac for excision by Cre to generate targeted alleles.

Endothelial-specific HSPA12B knockout mice were generated by cross-breeding the conditionally targeted HSPA12B mice with C57BL/6.Cg-Tg (Tek-cre) strain, which carries Cre recombinase under the control of the Tek promoter. Genotypes for the specific deletions were confirmed by PCR analysis of floxed allele (HspA12B-cko-1: gaagcaagcatattcatctcattactattc; HspA12B-cko-2: gcttgctcaaaagtgatggttgctc. 151 bp for knockout and 191 bp for WT mice), HSPA12B deletion (HspA12B-cko-1: gaagcaagcatattcatctcattactattc; HspA12B-cko-4: taaagcctacactcagatgagagcag, 240-bp product for deletion and > 2 kB or no product for WT control), and for Cre gene expression. Western blot and immunohistochemistry were also performed to identify endothelial-specific deficiency of HSPA12B.

### Immunofluorescence Staining

Immunohistochemistry was performed as described previously (22, 23). Briefly, the heart sections were stained with primary antibodies that are specific rabbit anti-HSPA12B and rat anti-CD31 (PECAM-1) (1:100) overnight at 4°C. The tissue sections were then incubated with secondary antibody Alexa Fluor-488 goat anti-rabbit immunoglobulin G (IgG) (H + L) (green; Thermo Fisher Scientific, Waltham, MA, United States) and Alexa Fluor-555 goat anti-rat IgG (H + L) (red) (Thermo Fisher Scientific) for 1 h at room temperature. The slides were examined with a fluorescent microscope at a magnification of 40×.

### Cecal Ligation and Puncture Polymicrobial Sepsis Model

Cecal ligation and puncture (CLP) was performed to induce polymicrobial sepsis in mice as previously described (22, 23). Briefly, the mice were anesthetized by isoflurane (induced by 5.0% and maintained by 1.5%). A midline incision was made on the anterior abdomen, and the cecum was exposed and ligated with a 4-0 suture. Two punctures were made through the cecum with an 18-gauge needle, and feces were extruded from the holes. The abdomen was then closed in two layers. Sham surgically operated mice served as the surgery control group. Immediately following surgery, a single dose of resuscitative fluid (lactated Ringer’s solution, 50 mL/kg body weight) was administered by subcutaneous injection (22, 23).

### Echocardiography

Transthoracic two-dimensional M-mode echocardiogram was obtained using a Toshiba Aplio 80 Imaging System (Toshiba Medical Systems, Tochigi, Japan) equipped with a 12-MHz linear transducer as described previously (22). M-mode tracings were used to measure LV end-systolic diameter and LV end-diastolic diameter. Percent fractional shortening (% FS) and ejection fraction (EF %) were calculated as described previously (22, 24).

### Accumulation of Neutrophils and Macrophages in the Myocardium

Accumulation of immune cells in heart tissues was examined with antineutrophil elastase antibody (Abcam, Cambridge, United Kingdom) and antimacrophage antibody F4/80 (1:50 dilution; Santa Cruz Biotechnology, Santa Cruz, CA, United States), separately (22, 23). Three samples from each group were evaluated, counterstained with hematoxylin, and examined with bright-field microscopy. Four different areas of each section were evaluated. The results are expressed as the numbers of neutrophils or macrophages per field examined with bright field microscope (40×).

### Myeloperoxidase Activity Assay

Myeloperoxidase (MPO) activity was measured using an MPO fluorometric Detection kit (Assay Designs Inc., Ann Arbor, MI, United States) according to the manufacturers’ instructions.

### Immunohistochemistry Staining

Immunohistochemistry was performed as described previously (22, 23). Briefly, heart tissues were immersion-fixed in 4% buffered paraformaldehyde, embedded in paraffin, and cut at 5-μm sections. The sections were stained with specific goat anti-intercellular adhesion molecule 1 (ICAM-1, 1:50 dilution; Santa Cruz Biotechnology) and rabbit anti-VCAM-1 (1:50 dilution, Santa Cruz Biotechnology), respectively, and treated with the ABC staining system (Santa Cruz Biotechnology) according to the instructions of the manufacturer. Three slides from each block were evaluated, counterstained with hematoxylin, and examined with bright field microscope (40×). Four different areas of each section were evaluated.

### Electrophoretic Mobility Shift Assay

Nuclear proteins were isolated from heart samples as previously described (22, 23). Nuclear factor κB (NF-κB) binding activity was performed using a LightShift Chemiluminescent EMSA (electrophoretic mobility shift assay) kit (Thermo Fisher Scientific) as described previously (22, 25) in a 20-μL binding reaction mixture containing 1× binding buffer, 50 ng poly dI:dC, 20 fmol of double-stranded NF-κB consensus oligonucleotide that was end-labeled with biotin, 15 μg nuclear proteins. The binding reaction mixture was incubated at room temperature for 20 min and analyzed by electrophoresis and then transferred to a nylon membrane. The biotin end-labeled DNA was detected using the streptavidin–horseradish peroxidase conjugate and the chemiluminescent substrate (22, 25).

### Enzyme-Linked Immunosorbent Assay for Cytokine Assay

The levels of cytokines [tumor necrosis factor α (TNFα), interleukin 6 (IL-6)] in cell-free supernatants were measured by enzyme-linked immunosorbent assay development kits (Peprotech, Rocky Hill, NJ, United States) according to manufacturers’ instructions as described previously (22, 23).

### Western Blot

Western blot was performed as described previously (22, 23). Briefly, the cellular proteins were separated by sodium dodecyl sulfate–polyacrylamide gel electrophoresis and transferred onto Hybond ECL membranes (Amersham Pharmacia, Piscataway, NJ, United States). The ECL membranes were incubated with the appropriate primary antibodies (anti–VCAM-1 and anti–ICAM1 from Santa Cruz Biotechnology; anti-CD63, anti-CD81, from System Biosciences, Palo Alto, CA, United States; anti-GAPDH from Meridian Life Science, Inc., Memphis, TN, United States, respectively. Anti-HspA12B is a kind gift from Dr. Han Zhihua) followed by incubation with peroxidase-conjugated secondary antibodies (Cell Signaling Technology, Inc., Danvers, MA, United States) and analysis by the ECL system (Amersham Pharmacia). The signals were quantified using the G: Box gel imaging system by Syngene (Syngene, USA, Fredrick, MD, United States).

### Isolation of Exosomes

Ten hours after CLP, the blood was collected from the experimental mice followed by centrifugation at 5,400 revolutions/min (rpm) for 15 min at 18°C. The supernatant was collected and added with ExoQuick exosome precipitation solution (63 μL/250 μL plasma, ExoQ5A-1; SBI, Palo Alto, CA, United states) according to manufacturer’s instruction.

### Isolation of RNA From Exosomes

Total RNA was extracted from the exosomes using Trizol (RN190; Molecular Research Center, Cincinnati, OH, United States) according to manufacturer’s instructions. Approximately 10 ng of total RNA was applied to examination of miRNA levels as described previously (26).

### Quantitative PCR Assay of MiRNAs

MicroRNAs were isolated from heart tissues or exosomes using the mirVana miR isolation kit (Ambion, Austin, TX, United States) as described previously (27). MicroRNA levels were quantified by quantitative PCR (qPCR) using specific Taqman assays (Applied Biosystems, Foster City, CA, United States) and specific primers (Applied Biosystems, primer identification numbers: 002228 for hsa–miR-126-3p and 001973 for snRU6). The levels of miRs were quantified with the 2(^–^ΔΔct) relative quantification method that was normalized to the U6 small nucleolar RNA (snRU6).

### Treatment of ECs With Exosomes

Exosomes were isolated from sham and septic mice using ExoQuickTC exosome precipitation solution (ExoQ5A-1; SBI). Human umbilical vein ECs were treated with exosomes (5 μg/mL) diluted in conditional medium, which was exosome-free medium prepared by centrifugation at 120,000 rpm for 18 h at 4°C. After treatment, HUVECs were collected for analysis of adhesion molecules ICAM-1 and VCAM-1 by Western blot (22, 23).

### Transfection of MiRNA Mimics Into ECs

Human umbilical vein ECs (1 × 10^6^) in six-well plates were transfected with 40 nmol of miR-126 mimics (Ambion), anti–miR-126 mimics (Exiqon) and miR-scrambled control (Exiqon), respectively by Lipofectamine 2000 (Thermo Fisher Scientific). Twenty-four hours after transfection, the cells were treated with LPS (1 μg/mL) for 24 h. The cells were harvested for analysis of adhesion molecules (VCAM-1 and ICAM-1) by Western blot.

### Preparation of Exosomal miR-126

Bone marrow stromal cells (BMSCs) were isolated from HSPA12B^–/–^ and WT mice as described previously (28). Briefly, mice were euthanized, and bone marrow was isolated by flushing the femur and tibia with Dulbecco modified Eagle medium (DMEM) using a 25-gauge 0.5-inch needle (BD, San Jose, CA, United States). The bone marrow was dissociated by syringe. Cell mixture was cultured in DMEM supplemented with 10% fetal bovine serum (FBS) (HyClone; Thermo Fisher Scientific), glutamine (2 mM), and penicillin/streptomycin (50 U/mL and 50 mg/mL; Sigma-Aldrich, St. Louis, MO, United States). After incubation at 37°C with 5% CO_2_, non-adherent cells were removed carefully by two washes with phosphate-buffered saline (PBS), and fresh medium was replaced. The medium was changed every other day. Cells at the fourth to seventh generation were transfected with 40 nmol/L hsa–miR-126 mimics (MC12841; Ambion), hsa–miR-126 inhibitor (MH12841; Ambion), or Cy3 dye–labeled miR-scrambled control (AM17010; Ambion), using Lipofectamine 2000 transfection reagent (Thermo Fisher Scientific) according to the manufacturer’s protocol. Twenty-four hours after transfection, supernatants were harvested for exosome isolation using Exoquick-TC Exosome Precipitation Solution (SBI) according to the manufacturer’s protocol.

### *In vivo* Delivery of Exosomal miR-126 Into Mouse Hearts

Mice were transfected with exosomes loaded with miR-126 or exosomes loaded with miR-control through the right carotid artery as described previously (27, 29). Briefly, mice were intubated and mechanically ventilated. The anesthesia was induced by 5% isoflurane and maintained by 1.5% isoflurane driven by 100% oxygen. Body temperature was maintained at 37°C by surface water heating. An incision was made in the middle of the neck, and the right common carotid artery was carefully exposed. A microcatheter was introduced into the isolated common carotid artery and positioned into the aortic root. Exosomes (10 μg diluted in 100 μL PBS) loaded with miR-126 or loaded with miR-Con were injected through the microcatheter immediately after the induction of polymicrobial sepsis. The microcatheter was gently removed, and the common carotid artery was tightened before the skin was closed (22, 23).

### Statistical Analysis

The data are expressed as mean ± SE. Comparisons of data between groups were made using one-way analysis of variance, and Tukey procedure for multiple-range tests was performed. The log-rank test was used to compare group survival trends. Probability levels of 0.05 or smaller were used to indicate statistical significance.

## Results

### EC-Specific Deficiency of HSPA12B (HSPA12B^–/–^) Results in Increased Mortality in Polymicrobial Sepsis

**FIGURE 1:**
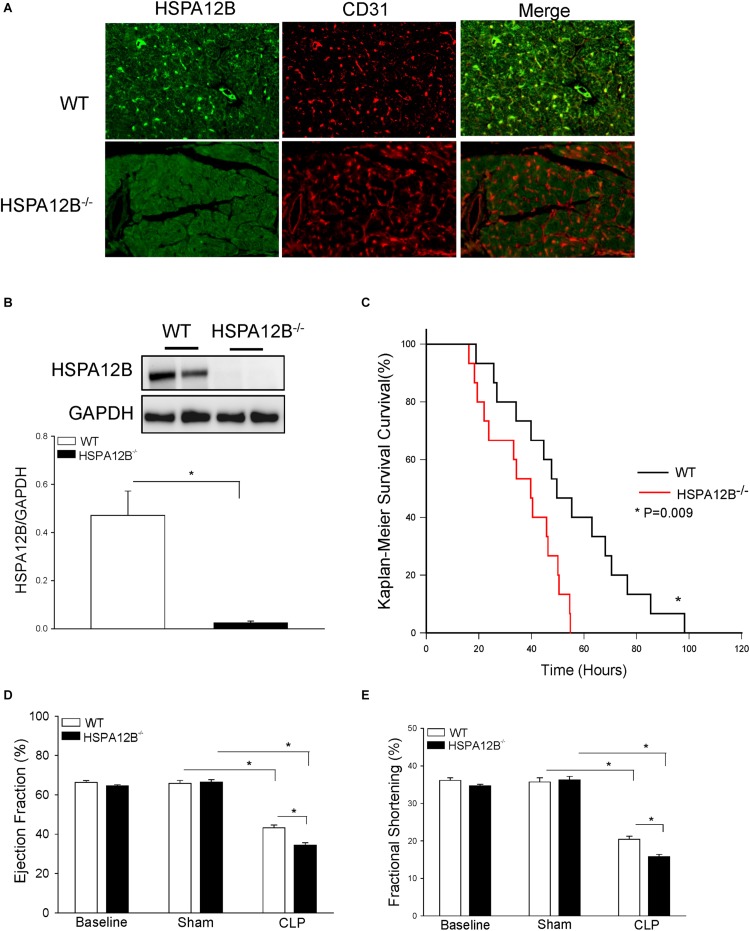
Endothelial-specific deficiency of HSPA12B results in increased mortality and worsened cardiac dysfunction in polymicrobial sepsis. **(A,B)** HSPA12B is expressed in the ECs of WT myocardium but not in HSPA12B^–/–^ mice. **(A)** Heart tissues from WT and HSPA12B^–/–^ mice were sectioned and subjected to immunostaining with anti-CD31 (EC marker) and anti-HSPA12B. There is a negative staining of HSPA12B in the myocardium of HSPA12B^–/–^ mice. The immunofluorescent staining was examined with fluorescent microscope (40×). **(B)** Western blot analysis of HSPA12B expression in the myocardium of WT and HSPA12B^–/–^ mice. **(C)** Sepsis increases the mortality of HSPA12B^–/–^ mice. Wild-type and HSPA12B^–/–^ mice were subjected to CLP sepsis. Sham surgical operation served as sham control. The survival rate was closely monitored up to 5 days (*n* = 15–16/group). **(D,E)** Cardiac function was examined by echocardiography before and 6 h after CLP (*n* = 6–13/group). Cecal ligation and puncture sepsis markedly decreases ejection fraction (EF %) and fractional shortening (FS %) in WT mice. However, the values of EF % and FS % in HSPA12B^–/–^ septic mice were further decreased compared with WT septic mice. **(D)** (EF %) and **(E)** (FS %). *P < 0.05 compared with indicated group.

We first examined the expression of HSPA12B in the myocardium. As shown in Figure 1A, HSPA12B is specifically expressed on cardiac ECs as evidenced by positive immunofluorescent staining of HSPA12B on ECs in the myocardium from WT mice but not from HSPA12B^–/–^ mice. Western blot analysis shows the high levels of myocardial HSPA12B in WT mice but not in HSPA12B^–/–^ mice (Figure 1B). Figure 1C shows that EC HSPA12B deficiency accelerates mortality of CLP septic mice. The time to 50% mortality in WT septic mice was 56 h, and 100% occurred at 100 h after induction of CLP-sepsis. In HSPA12B^–/–^ septic mice, however, the time to 50% mortality was 40 h. The mortality reached to 100% was 60 h after induction of CLP sepsis (*P* < 0.01). These data indicate that EC HSPA12B plays a role in reducing the mortality associated with polymicrobial sepsis.

### Endothelial HSPA12B Deficiency Results in Worsened Cardiac Dysfunction in Polymicrobial Sepsis

Cardiomyopathy is a major consequence of sepsis and contributes to mobility and mortality (30). Figures 1D,E show that CLP sepsis markedly decreased the values of EF% (34.3%) and % FS (42.8%) in WT septic mice and 48.2 and 56.5% in HSPA12B^–/–^ septic mice, when compared with the respective sham controls. HSPA12B^–/–^ septic mice exhibited a lower EF% (20.5%) and FS% (22.8%) than in WT septic mice. There was no significant difference in the baseline values of EF% and %FS between WT and HSPA12B^–/–^ mice. These data indicate that EC HSPA12B plays an important role in the regulation of cardiac function during polymicrobial sepsis.

### Inflammatory Cells Are Increased in the Myocardium of HSPA12B^–/–^ Septic Mice

**FIGURE 2:**
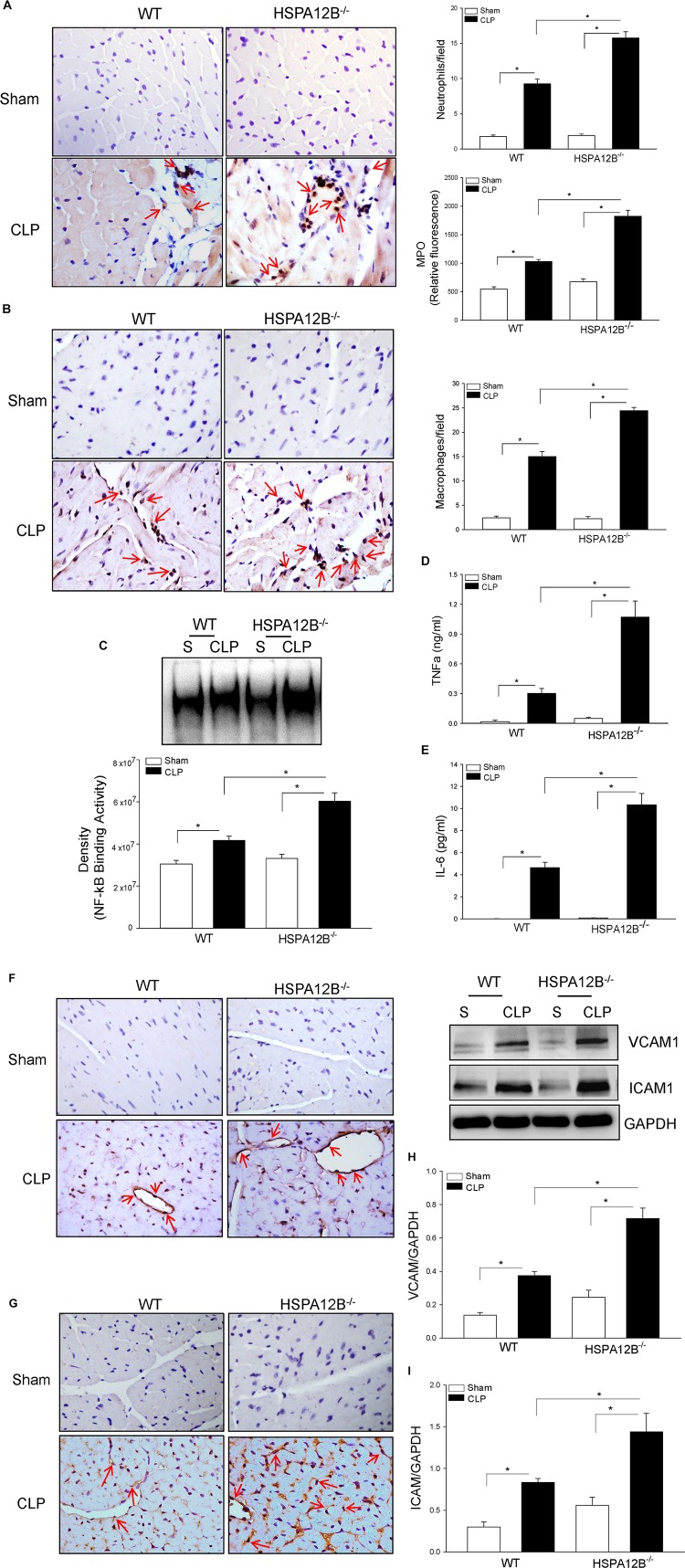
Increased accumulation of immune cells and NF-κB binding activity in the myocardium of HSPA12B^–/–^ septic mice. Wild-type and HSPA12B^–/–^ mice were subjected to CLP sepsis or sham surgical operation. Heart tissues were harvested 6 h after CLP. **(A)** The accumulation of neutrophils in heart tissues was examined by immunohistochemistry with antineutrophil antibody and MPO activity (*n* = 6–8/group). **(B)** Macrophages in the heart tissues were examined by antimacrophage antibody F4/80. The positive staining of neutrophils and macrophages are dark brown color marked with red arrows. **(C)** Myocardial NF-κB binding activity in WT and HSPA12B^–/–^ septic mice was performed with EMSA (*n* = 6–8/group). **(D,E)** Serum cytokine TNFα **(D)** and IL-6 **(E)** levels were measured by EMSA kits (*n* = 4–8/group). **(F–I)** Increased expression of adhesion molecules in the myocardium of WT and HSPA12B^–/–^ septic mice. Heart tissues were harvested 6 h after CLP, sectioned, and subjected to immunohistochemistry staining with anti–VCAM-1 **(F)** and anti–ICAM-1 **(G)** antibodies. **(H,I)** Western blot analysis of VCAM-1 **(H)** and ICAM1 **(I)** levels in the heart tissues. *n* = 4–8/group. *P < 0.05 compared with indicated groups. The immunohistochemistry staining was examined with bright field microscope (40×).

Increased accumulation of inflammatory cells, including neutrophils and macrophages, in the myocardium contributes to septic cardiomyopathy (22). Figure 2A shows that CLP sepsis markedly increased the numbers of neutrophils (9.3 ± 0.66 vs. 1.8 ± 0.22) and MPO activity (89.9%) in the myocardium of WT mice, when compared with WT sham controls. In contrast, neutrophil accumulation in the myocardium and myocardial MPO activity in HSPA12B^–/–^ septic mice was significantly 72 and 88% greater than in WT septic mice. Cecal ligation and puncture sepsis also significantly increased the numbers of macrophages (15.6 ± 1.01 vs. 2.6 ± 0.0.36) in the myocardium of WT mice compared with sham control (Figure 2B). In HSPA12B^–/–^ septic mice, macrophage accumulation in the myocardium was markedly 57.9% higher than in WT septic mice. The data indicate that EC HSPA12B could attenuate infiltration of immune cells into the myocardium during polymicrobial sepsis.

### Increased Myocardial NF-κB Activation and Serum Inflammatory Cytokine Levels in HSPA12B^–/–^ Septic Mice

Nuclear factor κB is an important transcription factor that regulates inflammatory cytokine production (31). Proinflammatory cytokines have been demonstrated to play a role in cardiovascular dysfunction during sepsis/septic shock (32). Figure 2C shows that myocardial NF-κB binding activity was markedly increased 36.8% in WT septic mice and 82.3% in HSPA12B^–/–^ septic mice, when compared with the respective sham controls. Cecal ligation and puncture sepsis also significantly increased the serum levels of TNFα (Figure 2D) and IL-6 (Figure 2E) in WT septic mice. However, the levels of serum TNFα and IL-6 in HSPA12B^–^/^–^ septic mice were markedly 243 and 223% greater than in WT septic mice (Figures 2D,E). The data indicate that EC HSPA12B plays a role in the regulation of NF-κB activation and proinflammatory cytokine production during polymicrobial sepsis.

### HSPA12B^–/–^ Results in Increased Expression of Adhesion Molecules Following Polymicrobial Sepsis

Increased expression of adhesion molecules on ECs promotes the infiltration of inflammatory cells into the myocardium (33). Figures 2F,G show that CLP sepsis increased the immunostaining of VCAM-1 (F) and ICAM-1 (G) in the myocardium of WT mice. However, there is more positive immunostaining for VCAM-1 and ICAM-1 in the myocardium from HSPA12B^–/–^ septic mice than in WT septic mice. Western blot analysis shows that CLP sepsis markedly increased the levels of myocardial VCAM-1 (Figure 2H) and ICAM-1 (Figure 2I) in WT mice. The levels of myocardial VCAM-1 and ICAM-1 in HSPA12B^–/–^ septic mice were further increased 173 and 191%, respectively, when compared with WT septic mice. The data suggest that HSPA12B is involved in the regulation of adhesion molecule expression on ECs, which ultimately facilitate the infiltration of inflammatory cells into the myocardium following polymicrobial sepsis.

### Increased HSPA12B Levels Suppress LPS-Induced VCAM-1 and ICAM-1 Expression in ECs

**FIGURE 3:**
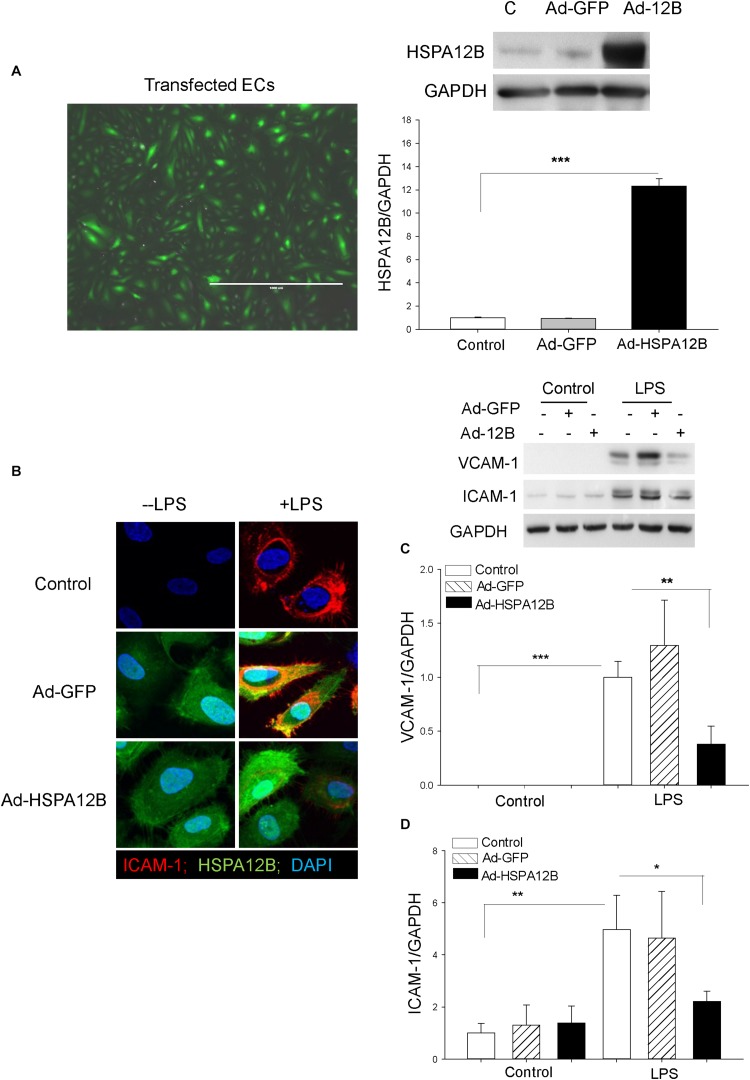
HSPA12B attenuates LPS-induced expression of adhesion molecules in ECs. Endothelial cells (HUVECs) were transfected with adenovirus-expressing HSPA12B (Ad-HSPA12B) or Ad-GFP 24 h before the cells were stimulated with LPS. **(A)** Green color indicates Ad-HSPA12B transfected into the HUVECs. Western blot shows that transfection of HUVECs with Ad-HSPA12B increased the levels of HSPA12B. **(B)** Confocal microscopy examination (66×) shows that Ad-HSPA12B transfection attenuates LPS-induced ICAM-1 (red color) expression in HUVECs. Green color indicates HSPA12B; blue color indicates nucleus stained with DAPI. **(C,D)** Western blot shows that Ad-HSPA12B transfection significantly suppressed LPS-induced VCAM-1 **(C)** and ICAM-1 **(D)** expression in HUVECs. *n* = 3/groups. *P < 0.05; **P < 0.01; ***P < 0.001 compared with indicated groups.

To further investigate the role HSPA12B in the regulation of adhesion molecule expression during polymicrobial sepsis, we performed *in vitro* experiments. Endothelial cells (HUVECs) were transfected with adenovirus expressing HSPA12B (Ad-HSPA12B) or Ad-GFP (Figure 3A). Twenty-four hours after transfection, the cells were stimulated with LPS (1 μg/mL) for 24 h. Confocal microscope examination shows that LPS stimulation increased ICAM-1 expression as evidenced by showing more immunofluorescent staining of ICAM-1 in LPS-stimulated ECs (Figure 3B). Western blot analysis also shows that LPS stimulation markedly increased the expression of VCAM-1 (Figure 3C) and ICAM-1 (Figure 3D), when compared with untreated control. However, both immunostaining and Western blot analysis show that increased HSPA12B expression by Ad-HSPA12B transfection markedly suppressed LPS-stimulated expression of VCAM-1 and ICAM-1. The data suggest that HSPA12B may prevent upregulation of adhesion molecule expression in ECs during sepsis.

### HSPA12B Upregulates MiRNA-126 Expression in ECs

**FIGURE 4:**
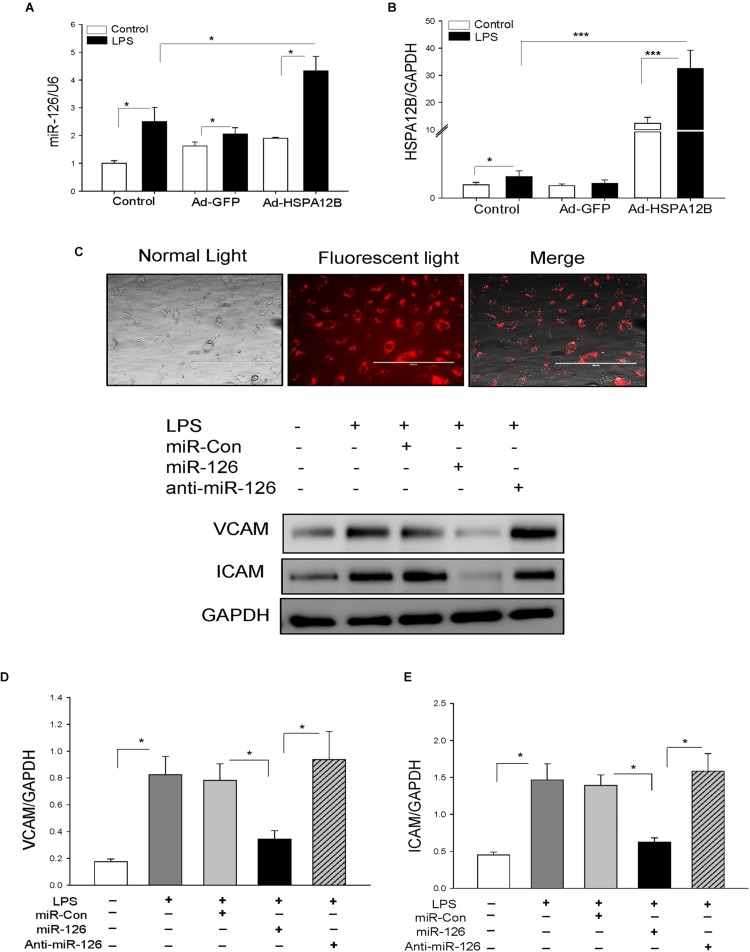
Transfection of endothelial cells with miR-126 mimics prevented LPS-induced expression of adhesion molecule expression. Endothelial cells (HUVECs) were transfected with 40 nmol of microRNAs (scrambled miR-control, miR-126 mimics, or anti–miR-126) by Lipofectamine 2000. Twenty-four hours after transfection, the cells were treated with LPS (1 μg/mL) for 6 h. **(A)** miR-126 levels and **(B)** HSPA12B expression. **(C)** Transfection of miR-126 mimics (red color) into endothelial cells. Transfection of miR-126 mimics suppresses LPS-induced expression of VCAM-1 **(D)** and ICAM1 **(E)** in endothelial cells. *n* = 3–4/group. *P < 0.05 compared with indicated groups.

MicroRNA-126 is predominantly expressed in ECs and suppresses adhesion molecule expression (18, 20). We examined whether HSPA12B suppressed LPS-induced adhesion molecule expression is mediated via upregulation of miR-126 expression in ECs. We transfected HUVECs with Ad-HSPA12B or Ad-GFP, which served as vector control. Twenty-four hours after transfection, the cells were stimulated with LPS. The levels of miR-126 were measured by qPCR. As shown in Figure 4, LPS stimulation increased the levels of miR-126 (A) and HSPA12B (B) in ECs. Interestingly, LPS stimulation further increased expression of miR-126 and HSPA12B after the cells were transfected by Ad-HSPA12B. The data indicate that HSPA12B is involved in the regulation of miR-126 expression in ECs following LPS challenge.

### MiR-126 Suppresses LPS-Increased Adhesion Molecule Expression in ECs

We then examined whether increased miR-126 levels will suppress LPS-stimulated adhesion molecule expression in ECs. We transfected ECs with miR-126 mimics or miR-control mimics 24 h before the cells were stimulated with LPS. Figure 4C shows a high efficiency of the miRNA transfection into ECs. Transfection of ECs with miR-126 mimics prevented LPS-stimulated the expression of VCAM-1 (Figure 4D) and ICAM-1 (Figure 4E). Antagomir-126 or miR-control mimics transfection did not alter LPS-stimulated increases in the expression of adhesion molecules. The data suggest that miR-126 targets adhesion molecule expression in ECs.

### Decreased Levels of miR-126 Levels in Serum Exosomes From HSPA12B^–/–^ Septic Mice

**FIGURE 5:**
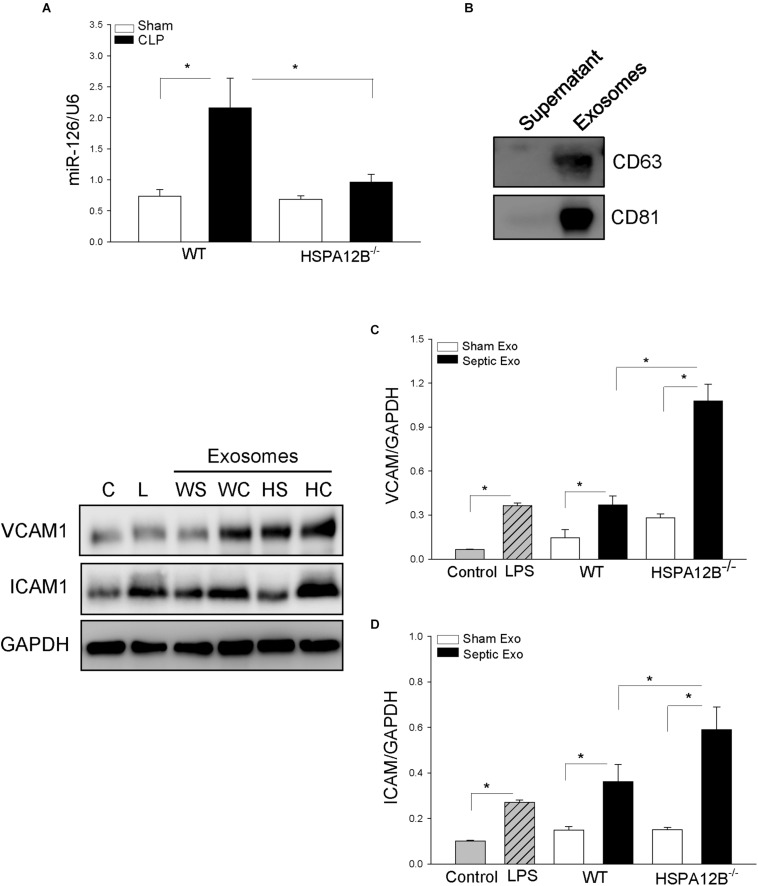
Serum exosomes from HSPA12B^–/–^ septic mice increased expression of adhesion molecules in endothelial cells. Wild-type and HSPA12B^–/–^ mice were subjected to CLP sepsis or sham surgical operation. Ten hours after CLP, blood was collected for the isolation of serum exosomes with ExoQuick exosome isolation kit. **(A)** miR-126 levels in the isolated serum exosomes were examined by qPCR. **(B)** Exosome markers (CD63 and CD81). **(C)** Endothelial cells (HUVEC) were treated with isolated exosomes for 12 h. The cells were harvested for analysis of VCAM-1 **(C)** and ICAM1 **(D)** by Western blot (*n* = 4–6/group). C, control; L, LPS; WS, WT sham; WC, WT CLP; HS, HSPA12B^–/–^ sham; HC, HSPA12B^–/–^ CLP; Exo, exosomes. ^∗^*P* < 0.05 compared with indicated groups.

To investigate whether increased adhesion molecule expression in the myocardium from HSPA12B^–/–^ septic mice will be associated with miR-126 levels, we collected serum from WT and HSPA12B^–/–^ sham and septic mice, isolated exosomes, and examined miR-126 levels with qPCR. As shown in Figure 5A, CLP sepsis markedly increased the levels of miR-126 in exosomes from WT mice but not from HSPA12B^–/–^ mice. The levels of miR-126 in HSPA12B^–/–^ septic exosomes were significantly lower than in WT septic exosomes. The data indicate that lower levels of miR-126 in HSPA12B^–/–^ septic exosomes may be responsible for increased expression of adhesion molecules and accumulation of immune cells in the myocardium of HSPA12B^–/–^ septic mice.

### HSPA12B^–/–^ Septic Exosomes Enhance the Expression of Adhesion Molecules on *EC*s

To investigate the role of HSPA12B^–/–^ septic exosomes in adhesion molecule expression on ECs, we collected blood and isolated serum exosomes from WT and HSPA12B^–/–^ mice. We then treated ECs with the isolated exosomes and examined the levels of adhesion molecules. Figure 5B shows exosome markers (CD63 and CD81) in the isolated exosomes. Figures 5C,D shows that treatment of ECs with WT septic exosomes markedly increased 87.2% VCAM-1C and 157.3% ICAM-1D levels, when compared with the WT sham exosome-treated group. However, treatment of ECs with HSPA12B^–/–^ septic exosomes resulted in greater levels of VCAM-1 and ICAM-1, when compared with WT septic exosome treatment. The levels of VCAM-1 and ICAM-1 in the HSPA12B^–/–^ septic exosomes group were 75.1 and 78.9% greater than in WT septic exosome-treated cells. The data suggest that lower levels of miR-126 in the exosomes from HSPA12B^–/–^ septic mice may be responsible for increased adhesion molecule expression in the myocardium during polymicrobial sepsis.

### Delivery of miR-126 Carried by Exosomes Suppressed Adhesion Molecule Expression in the Myocardium From HSPA12B^–/–^ Septic Mice

**FIGURE 6:**
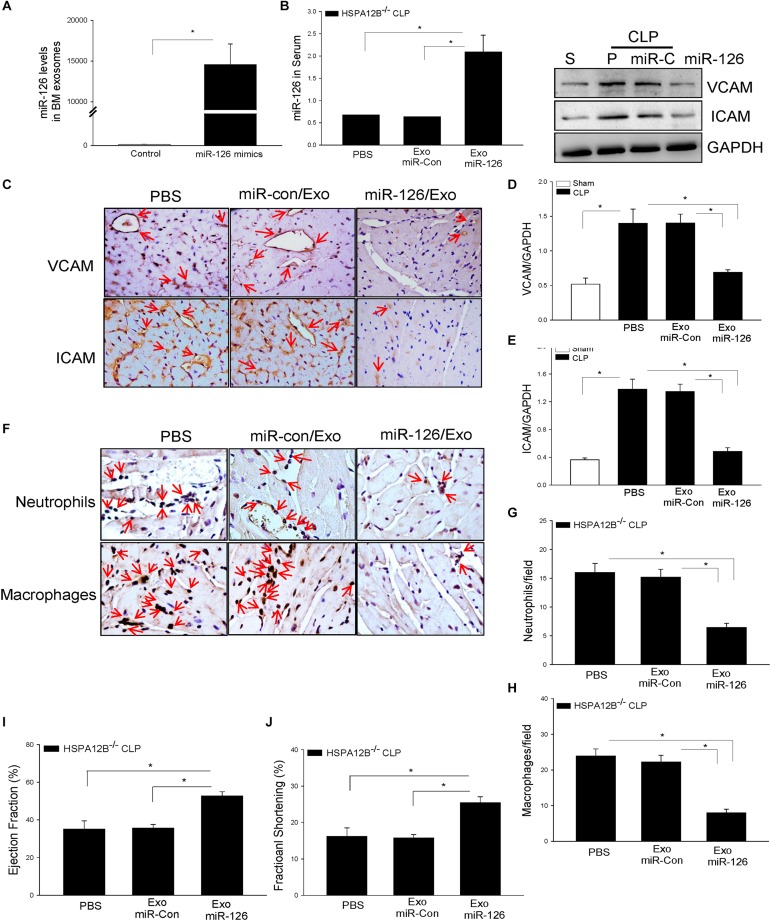
MicroRNA-126 carried by exosomes derived from bone marrow stromal cells suppressed adhesion molecule expression in the myocardium of HSPA12B^–/–^ septic mice. Bone marrow stromal cells (BMSCs) were isolated from HSPA12B^–/–^ mice and transfected with 40 nmol/L scrambled miR-control or miR-126 mimics at the fourth to seventh generation. Twenty-four hours after transfection, exosomes were isolated from supernatants. **(A)** The levels of miR-126 in exosomes were examined qPCR. (B) HSPA12B^–/–^ mice were transfected with exosomes loaded with miR-126 mimic or scrambled miR-control through the right carotid artery immediately before induction of CLP sepsis. Serum miR-126 levels were examined by qPCR 6 h after CLP. **(C)** VCAM-1 and ICAM1 expressions in the heart tissues were examined by immunohistochemistry staining with anti–VCAM-1 and anti–ICAM-1 antibodies. **(D,E)** Western blot analysis of VCAM-1 and ICAM1 levels in the myocardium of HSP12B^–/–^ septic mice (*n* = 6–9/group). **(F)** The accumulation of neutrophils and macrophages in the myocardium was examined by immunohistochemistry with antineutrophil elastase antibody and antimacrophage antibody F4/80. **(G,H)** miR-126 carried by exosomes decreased the numbers of neutrophils **(G)** and macrophages **(H)** in the myocardium of HSPA12B^–/–^ septic mice. **(I,J)** miR-126 carried by exosomes improves cardiac function (EF %, FS %) measured by echocardiography. *n* = 6–9/group for Western blot and *n* = 3/group for immunohistochemistry. *P < 0.05 compared with indicated groups. The immunohistochemistry staining was examined with bright field microscope (40×).

To further investigate the role of exosomal miR-126 in the regulation of adhesion molecule expression, we isolated BMSCs from HSPA12B^–/–^ mice, transfected BMSCs with miR-126 mimics or miR-control mimics, and isolated exosomes from cultured medium. Figure 6A shows that miR-126 levels in the exosomes were significantly greater than in the exosomes loaded with miR-control. We delivered the exosomal miR-126 or exosomal miR-control into the myocardium through the right carotid artery (27, 29) immediately after induction of CLP in HSPA12B^–/–^ mice. Twenty-four hours after delivery, we collected blood and measured serum levels of miR-126 levels by qPCR. Figure 6B shows that delivery of exosomal miR-126 markedly increased the serum miR-126 levels (178%) in HSPA12B^–/–^ septic mice compared with delivery of exosomal miR-control. Immunostaining shows that delivery of exosomal miR-126 prevented sepsis-increased expression of VCAM-1 and ICAM-1 (Figure 6C). Western blot analysis shows that exosomal miR-126 transfection markedly prevented sepsis-induced increases in VACM-1 (Figure 6D) and ICAM-1 (Figure 6E) levels in the myocardium. We also analyzed the effect of delivery of exosomal miR-126 on sepsis-induced accumulation of immune cell in the myocardium. As shown in Figure 6F, delivery of exosomal miR-126 significantly decreased sepsis-induced accumulation of neutrophil (Figure 6G) and macrophage (Figure 6H) in the myocardium from HSPA12B^–/–^ septic mice. The data clearly suggest that decreased miR-126 levels could be responsible for increased expression of adhesion molecules and immune cell accumulation in the myocardium in HSPA12B^–/–^ septic mice.

### Delivery of Exosomal miR-126 Improved Cardiac Function in HSPA12B^–/–^ Septic Mice

We then examined whether suppression of adhesion molecule expression and reduced accumulation of immune cells in the myocardium by exosomal miR-126 will improve cardiac function in HSPA12B^–/–^ septic mice. Figures 6I,J show that delivery of exosomal miR-126 into the myocardium of HSPA12B^–/–^ septic mice significantly increased the values of EF% (47.8%) and %FS (61.2%) respectively, when compared with the exosomal miR-control group. The data demonstrated that miR-126 plays an important role in cardiac function by suppressing adhesion molecule expression during polymicrobial sepsis.

## Discussion

The present study has shown that EC-specific HSAP12B exerts a protective role in polymicrobial sepsis–induced cardiomyopathy. There are several important findings in the present study. First, deficiency of endothelial-specific HSPA12B (HSPA12B^–/–^) results in severe cardiac dysfunction and poor survival outcome following polymicrobial sepsis, suggesting that endothelial HSP12B serves a protective role in cardiac function in sepsis. Second, endothelial HSPA12B deficiency promotes the increased expression of adhesion molecules and leads to accumulation of immune cells in the myocardium. This indicates that HSPA12B is involved in controlling adhesion molecule expression and immune cell infiltration into the myocardium following CLP sepsis. Third, the serum exosomes isolated from HSPA12B^–/–^ septic mice contain lower levels of miR-126 when compared with the exosomes from WT septic mice. MicroRNA-126 specifically targets adhesion molecules (20). Therefore, it is possible that lower levels of miR-126 may be responsible for increased adhesion molecule expression and accumulation of immune cells in the myocardium. Finally, we loaded miR-126 onto exosomes derived from HSPA12B^–/–^ BMSCs, delivered into the myocardium of HSPA12B^–/–^ septic mice, and observed that transfection of miR-126 carried by exosomes significantly improves cardiac function of HSPA12B^–/–^ septic mice by suppressing the expression of adhesion molecules and decreasing the infiltration of inflammatory cells into the myocardium. The data suggest that endothelial miR-126 plays an important role in HSPA12B regulation of adhesion molecule expression during polymicrobial sepsis.

It is well known that EC dysfunction contributes to the pathophysiology of sepsis/septic shock and multiple organ dysfunction (34). Biomarkers of EC dysfunction have been concentrated on ICAMs. Increased expression of adhesion molecules, such as ICAM-1 and VCAM-1, has been shown to recruit neutrophils and macrophages into the myocardium, leading to cardiac dysfunction in sepsis (35, 36). Activated macrophages also release chemokines to attract neutrophils into the myocardium (35). We have previously reported that polymicrobial sepsis (22) and endotoxemia (13) significantly increased the expression of adhesion molecules, resulting in accumulation of neutrophils and macrophages in the myocardium. Therefore, suppression of adhesion molecule expression could be an important approach for the attenuation of sepsis-induced cardiomyopathy. Indeed, we observed in our previous studies that transgenic mice with EC-specific expression of HSPA12B show a significant attenuation of endotoxin-increased adhesion molecule expression and cardiac dysfunction through activation of PI3K/Akt signaling (13). However, the precise mechanisms by which HSPA12B is required for EC function are still unsolved.

We observed in the present study that HSPA12B^–/–^ septic mice exhibit higher levels of adhesion molecules and greater immune cell accumulation in the myocardium than in WT septic mice. Our observation is consistent with previous studies showing HSPA12B is essential for EC functioning during sepsis/septic shock (13). Increased expression of ICAM-1 and VCAM-1 facilities the recruitment of macrophages and neutrophils into the myocardium, leading to inflammatory response in sepsis (35, 36). We observed that myocardial NF-κB binding activity and serum inflammatory cytokine levels, such as TNFα and IL-6, in HSPA12B^–/–^ septic mice were markedly greater than in WT septic mice. This suggests that endothelial HSPA12B may play a role in not only regulating adhesion molecule expression but also controlling NF-κB mediated inflammatory cytokine production in polymicrobial sepsis.

To investigate how EC deficiency of HSPA12B increased adhesion molecule expression and the accumulation of immune cells in the myocardium following polymicrobial sepsis, we examined the effects of serum exosomes isolated from experimental mice on adhesion molecule expression in ECs *in vitro*. Exosomes are membranous nanovesicles (30–100 nm), which arise inside many cells from endosomal compartments called multivesicular bodies (37). Recent evidence demonstrated that exosomes play a critical role in cell-to-cell communication and serve as a novel vehicle for transferring proteins and/or miRs (37–39) from one cell to another through membrane fusion with the target cells, by binding with specific receptors at the cell surface of target cells, or endocytotic internalization. Exosomes are also shown to play a key role in host immunity to pathogens during infection (40). Interestingly, we found that treatment of ECs with exosomes that were isolated from HSPA12B^–/–^ septic mice significantly increased the expression of VCAM-1 and ICAM1, when compared with WT septic exosomes. Our observations suggest that exosomes isolated from septic mice play a critical role in mediating EC dysfunction during sepsis.

At present, we do not understand which cells released exosomes into the serum in response to polymicrobial sepsis/shock. We also do not understand which compositions in the septic exosomes will be responsible for causing EC dysfunction. Interestingly, we observed that the levels of miR-126 in HSPA12B^–/–^ septic exosomes were significantly lower than in WT septic exosomes. MicroRNA-126 is predominately expressed in ECs (18), targets adhesion molecules (20), and regulates angiogenesis (19). Lower levels of miR-126 in the exosomes may be responsible for the higher levels of myocardial adhesion molecules of HSPA12B^–/–^ septic mice. To evaluate our hypothesis, we treated ECs with exosomes that were isolated from septic mice and observed that the levels of adhesion molecules in HSPA12B^–/–^ septic exosome-treated ECs were significantly greater than in the effect of WT septic exosomes. To confirm our observation, we transfected ECs with miR-126 mimics before the cells were treated with LPS and observed that transfection miR-126 markedly suppresses LPS-induced increases in the expression of adhesion molecules.

Our *in vitro* data indicate that suppression of adhesion molecule expression by miR-126 mimics may decrease the infiltration of inflammatory cells into the myocardium and result in attenuation of cardiac function *in vivo* following polymicrobial sepsis. To test this hypothesis, we isolated BMSCs from HSPA12B^–/–^ mice, transfected BMSCs with miR-126 mimics and isolated exosomes derived from BMSCs. We then delivered miR-126 carried by exosomes into the myocardium immediately after induction of polymicrobial sepsis. We observed that delivery of miR-126 carried by exosomes significantly improved cardiac function in HSPA12B^–/–^ septic mice. Importantly, delivery of exosomes loaded with miR-126 attenuated sepsis-induced expression of adhesion molecules and accumulation of macrophages and neutrophils in the myocardium of HSPA12B^–/–^ mice. At the moment, we do not understand the mechanisms by which endothelial-specific deficiency of HSPA12B results in lower levels of miR-126 in serum exosomes. However, our data suggest that targeting adhesion molecules is an important approach for maintenance of EC function and attenuation of inflammatory cell infiltration in the myocardium during polymicrobial sepsis. Exosomes loaded with miR-126 could be a novel approach for this purpose.

## Data Availability Statement

All datasets generated for this study are included in the article/Supplementary Material.

## Ethics Statement

The animal study was reviewed and approved the ETSU University Committee on Animal Care.

## Author Contributions

XZ performed experiments and wrote the manuscript. XW performed the miR-126 experiments. MF performed the miR and adhesion molecule expression experiments. FT and KY performed the in vitro experiments. TH performed the gene type identification experiments. LL interpreted the data and contributed to the discussion. JK was involved in the data statistical analysis. DW was involved in the data discussion and preparation of the manuscript. CL was involved in the experimental design, data interpretation, and preparation of the manuscript.

## Conflict of Interest

The authors declare that the research was conducted in the absence of any commercial or financial relationships that could be construed as a potential conflict of interest.

## Supplementary Material

FIGURE S1 The targeting strategy for the development of Flox/Flox mice.
