# Supplementary figures and images for: Endothelial HSPA12B Exerts Protection Against Sepsis-Induced Severe Cardiomyopathy via Suppression of Adhesion Molecule Expression by miR-126
Source: Front Immunol. 2020 Apr 29;11:566. doi: 10.3389/fimmu.2020.00566 (PMC7201039; doi:10.3389/fimmu.2020.00566)

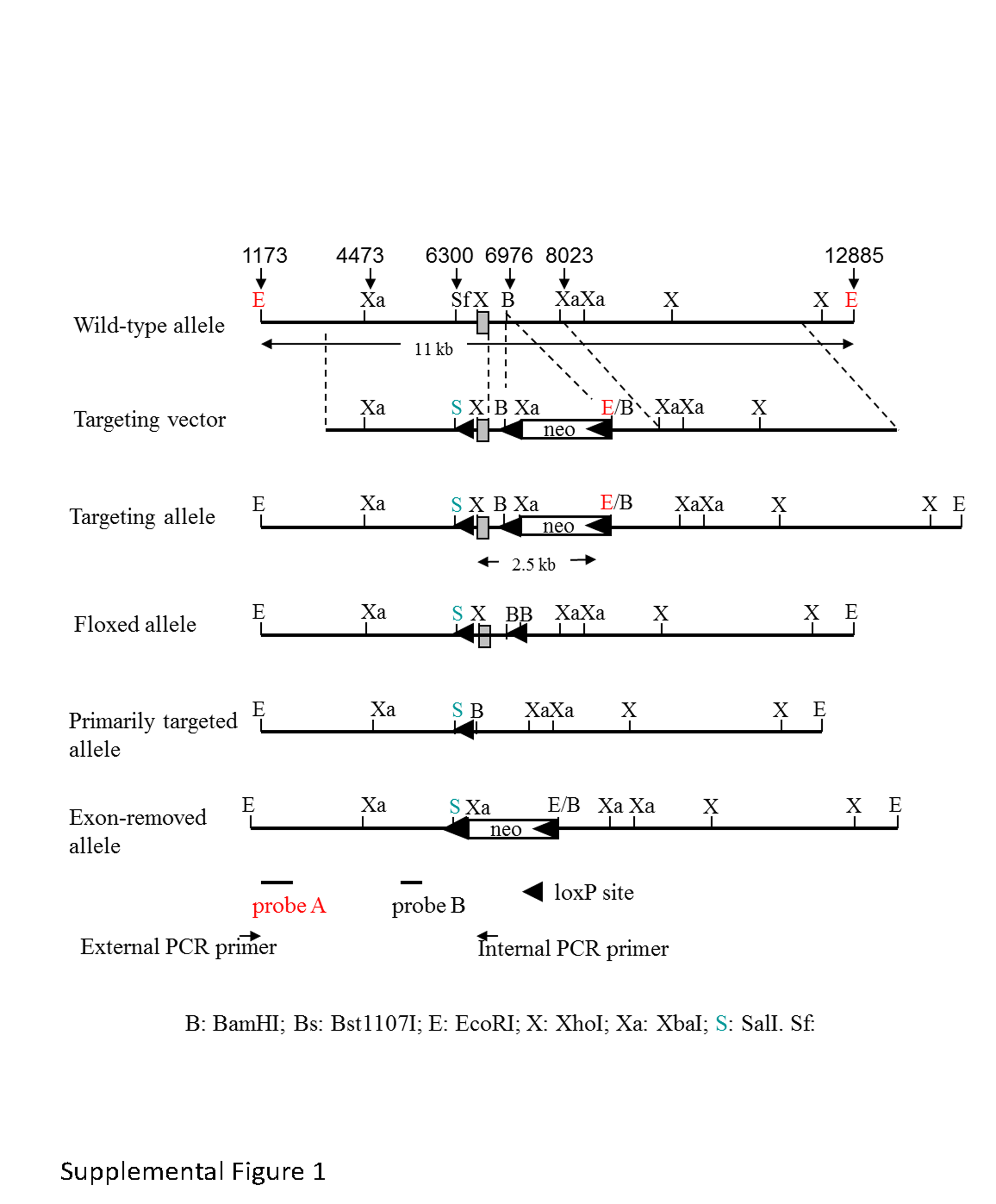

Supplement: FIGURE S1 — The targeting strategy for the development of Flox/Flox mice. [file Image_1.TIF]
